# Disaster displacement and zoonotic disease dynamics: The impact of structural and chronic drivers in Sindh, Pakistan

**DOI:** 10.1371/journal.pgph.0000068

**Published:** 2021-12-08

**Authors:** Dorien H. Braam, Rafiq Chandio, Freya L. Jephcott, Alex Tasker, James L. N. Wood

**Affiliations:** 1 Disease Dynamics Unit, Department of Veterinary Medicine, University of Cambridge, Cambridge, United Kingdom; 2 Department of Economics, University of Sindh, Sindh, Pakistan; 3 Department of Anthropology, University College London, London, United Kingdom; Mzuzu University, MALAWI

## Abstract

Projected increases in human and animal displacement driven by climate change, disasters and related environmental degradation will have significant implications to global health. Pathways for infectious disease transmission including zoonoses, diseases transmitted between animals and humans, are complex and non-linear. While forced migration is considered an important driver for the spread of zoonoses, actual disease dynamics remain under researched. This paper presents the findings of a case study investigating how disaster displacement affected zoonotic disease transmission risk following the 2010 ‘superfloods’ in Sindh province, Pakistan. We interviewed 30 key informants and 17 household members across 6 rural communities between March and November 2019, supported by observational studies and a review of secondary data. Results were analysed using the ecosocial theoretical framework. Buffalo, cattle and goats were often the only moveable asset, therefore livestock was an important consideration in determining displacement modality and destination location, and crowded locations were avoided to protect human and animal health. Meanwhile however, livestock was rarely included in the humanitarian response, resulting in communities and households fragmenting according to the availability of livestock provisions. We found that rather than a driver for disease, displacement acted as a process affecting community, household and individual zoonotic disease risk dynamics, based on available resources and social networks before, during and after displacement, rooted in the historical, political and socio-economic context. We conclude that in rural Sindh, disaster displaced populations’ risk of zoonoses is the result of changes in dynamics rooted in pre-existing structural and chronic inequalities, making people more or less vulnerable to disease through multiple interlinked pathways. Our findings have implications for policy makers and humanitarian responders assisting displaced populations dependent on livestock, with a call to integrate livestock support in humanitarian policies and responses for health, survival and recovery.

## 1. Introduction

The risk of zoonoses, diseases transmissible between animals and humans, depends on complex interactions between biological, environmental, socio-economic, political, technological factors [1, 2]. These determinants of health are increasingly considered in epidemiological research beyond reductionist characterisations of health-disease processes. Human and animal movement, in particular unplanned and forced migration, is often named one of these determinants [3–8]. The breakdown of health services, malnutrition, movement into new ecological zones, and subsequent crowding, poor shelters and sanitation increase the risk of infectious disease transmission, with exhaustion, malnutrition, stress arising from displacement further increasing disease susceptibility in animal and human populations [9–11]. Concern for zoonoses has resulted in the exclusion of ruminant livestock species in formal relief camps, affecting people’s livelihoods, resilience and recovery [12], with livestock support and veterinary public health rarely considered and funded within humanitarian responses. As most population displacement takes place in countries and communities where livestock plays an essential role in people’s livelihoods and food security [13], the lack of consideration of animals in displacement has severe short- and long-term consequences, exacerbating poverty and ill health [14].

Epidemiological research in humanitarian emergencies has largely remained focused on health outcomes and disease data, ignoring structural and contextual drivers [15]. Patterns of morbidity and mortality are linked to political and socio-economic inequities, which may be exacerbated or mitigated by formal and informal mechanisms and structures [16]. These mechanisms and structures, which often arise from (colonial) histories and patriarchal systems, compounded by macro-sociological institutional arrangements, and economic systems, are largely ignored [17, 18]. Occupying an undefined legal space, Internally Displaced Persons (IDPs) are particularly subjected to structural violence in the form of discrimination or exploitation, while facing systemic and structural obstacles to healthcare or other services [19]. During displacement, people may be forced to live on marginal land or in substandard housing [20], settings which exacerbate the spread of infectious diseases [21].

Traditional epidemiological outbreak narratives associate vulnerability with the probability that individual becomes ill within a given period [22], In this study we expand this definition to consider ‘vulnerability’ as a condition determined by physical, social, economic and environmental risk factors or processes, that increase the susceptibility of an individual or a community to be harmed by the impacts of zoonotic pathogens [23]. Vulnerability to zoonotic disease depends not only on the presence of pathogens, but on a variety of community and individual level characteristics, including the availability and distribution of resources, social status, connections, and health needs, which are influenced by socioeconomic and demographic factors determined through colonization, development, health policies, or other structural inequalities [22, 24–26].

This paper builds on the recent theoretical critiques of the social determinants of health, using an ecosocial theoretical framework for a systematic integrated analysis to consider drivers behind differences in zoonotic disease risk during displacement, more specifically acknowledging the societal arrangements of power and resources [27]. Ecosocial theory, first developed by Krieger in 1994, provides a framework for the inclusion of social theory and determinants of health to highlight and address disease distribution based on a systems approach [27–30]. Using its concepts, we can analyse dynamic and interacting micro- and macro-level processes in individual and population health. Krieger (2001) argues that non-contextual description of social determinants hampers understanding of causes of disease. Rather than focusing on the negative health outcomes of displacement, such as overcrowding and unhygienic living conditions, these can be framed as biological expressions of pre-existing and structural inequalities, rooted in the historical, political and socio-economic context. We use this framework to explore a case study of the 2010 ‘superfloods’ in Sindh province in Pakistan. Lacking comprehensive animal and human health data, we do not compare individual and population health differences, rather we use ecosocial concepts to discuss the influence of displacement to risks and pathways to zoonotic disease, with the aim to highlight areas for humanitarian and policy interventions. Through the ecosocial theoretical approach we consider interlinkages and factors which do not always allow for specific quantitative risk measurement, rather creating a ’web of causation’ considering multilevel risk factors [29].

## 2. Methods

We employed a case study methodology as the most relevant approach to answer an open research question about a complex contemporary phenomenon within a real-life context [31]. The case study was explored using semi-structured key informant and household interviews with community observations, supported by a review of available secondary qualitative and quantitative data drawn from both open and subscription databases, including data obtained from key informants during interviews. All data was analysed using a thematical analysis methodology.

### 2.1 Study area and participants

Sindh province covers three geographically distinct zones: the mountainous Kirthar Range in the west, the central alluvial Indus river plain, and the eastern Thar desert region extending into India, with an estimated total population of 48 million people, 51 percent of which is considered rural [32]. The province has a subtropical climate, consisting of hot summers and unpredictable rain patterns during monsoon season in July and August. The Indus river, after which the province is named, is central to Sindh culture and livelihoods, supplying an extensive network of irrigation for agriculture [33, 34]. The province is an important source of food supply to the rest of the country, with over 7.6 million acres of cultivated land for cash and food crops, with the highest cultivation density in the north and along the Indus [32]. Sindh is a primary producer of fruits and vegetables suitable to its climate, including dates and tomatoes, and is the second largest producer of staple crops after the province of Punjab [32]. Livestock contributes over 55 percent to Sindh’s agriculture sector, with cattle, buffalo, goats and sheep the main animals kept [32]. Rural livestock keepers derive up to 40 percent of their income from livestock, with the majority keeping less than four animals [35]. For our study, we focused on Thatta and Sujawal districts, based on their high disaster risk according to the Pakistan National Disaster Management Authority (NDMA), and high livestock dependency. Home to the Indus Delta, low-lying areas within these districts are regularly flooded, including during the nationwide 2010 superfloods, one of the most destructive disasters the country ever experienced in terms of damages and displacement.

We selected individuals for semi-structured key informant interviews using purposive and respondent driven sampling based on their technical, process and interpretive knowledge and expertise in public health, veterinary health and/or disaster displacement response, across government, United Nations (UN), non-profit and community-based organisations.

In addition, we conducted semi-structured household interviews and observational studies in selected communities across the districts, which had been displaced during the 2010 superfloods. Local staff members from the National Rural Support Programme (NRSP) selected six communities across the districts, which had been displaced during the 2010 superfloods. In each community, we interviewed at least two households, except in one community where a death during the research period prevented us from returning. The communities included in the study consisted of 40 to 250 households, clustered into patrilineal cultural groups. Households–defined by the Pakistan Bureau of Statistics as those sharing kitchen facilities—consisted of 5–12 members, usually parents and their children. Where grandparents live in the same house, these were counted as part of the household. Households (n = 12) were purposefully sampled based on socio-economic status reflected in livestock ownership (buffalo, cattle and/ or goats), and retainment before and after displacement, and semi-structured interviews were conducted with those adult household members responsible for animal husbandry and/ or livestock product processing, both male (n = 10) and female (n = 7), with both male and female household members interviewed in 5 of the sampled households.

### 2.2 Data collection

The semi-structured questionnaires focused on demographic and socio-economic characteristics, experiences of displacement, human and animal health and health-seeking practices, and were adjusted based on the individual’s role and experience in their field, in particular during the 2010 superfloods disaster. A key characteristic of semi-structured interviews is that these are flexible in the sense that participant respondents can refocus the interview based on the areas they feel most relevant. Questionnaires were developed based on secondary data review in collaboration with the Planning and Development Department Research and Training Wing in Sindh, and refined iteratively during the interviews. The questionnaires are available as ‘interview guides’ as S1 File.

Key informant interviews were conducted in the federal capital Islamabad, provincial capital Karachi and district capitals Hyderabad, Thatta and Sujawal between March–November 2019, at days and times chosen by the respondents.

Household interviews were conducted in October–November 2020. The interviews followed semi-structured questionnaires with questions focusing on demographic and socio-economic characteristics, experiences of displacement, human and animal health and health-seeking practices, using a multisite design to improve external validity [36].

To counter gender-based barriers to data collection, the lead researcher was supported by both a female and male translator. Data collection was conducted in respondents’ indigenous language Sindhi. Interviews were recorded after approval from the respondent, and transcribed into English. Household interviews were conducted during daytime due to security and resource constraints. Due to the communal household settings in Sindh, most interviews were conducted with more than one individual present. Observations within and around the household and community were made and recorded through photos and field notes to support contextualization and triangulation of the responses. The validity and reliability of data was strengthened by regular cross-checking responses with participants [36]. Following the qualitative research principle of data saturation, we continued interviewing until no new data and concepts were introduced, using a flexible approach for applied inductive thematic analyses [37, 38].

### 2.3 Ethical considerations

The study protocol was approved by the Human Biology Ethics Committee at the University of Cambridge (protocol number HBREC.2019.25) and in Sindh by the Planning and Development Department, Research and Training Wing of the Government of Sindh, which provided all required permits and approvals for conducting research. Information and informed consent letters were available in English and Sindhi. Participants were informed about the study during face-to-face recruitment, by telephone and/or e-mail, and informed about the voluntary bases of participation at the start of data collection. Copies of the study protocol and consent forms were available and shared with participants upon request. Key informants and household participants gave written consent, or verbal for illiterate respondents and where participants considered signing the consent form a potential risk to their anonymity. Verbal consent was recorded on a digital recorder before the start of the interview. for data collection and recording of interviews. Verbal consent was approved under HBREC.2019.25 as the study presented no more than minimal risk of harm to respondents. To preserve participants’ anonymity the analysis and discussion do not include any personal identifiable information.

To minimize the risk of exploitation and damaging research practices among vulnerable and marginalized community participants, we avoided discussing traumatizing events around the displacement experience by using semi-structured rather than closed questionnaires allowing participants to refocus discussions. We focused on communities who received support from NRSP to minimise risks of extractive research. The power imbalance between researcher and participant was mitigated as much as possible by the selection of households and individual participants through local NRSP staff, who are members of the respondents’ communities.

### 2.4 Data analysis

Interview and secondary data was coded manually in English, and themes constructed from the data using a thematical analysis approach [39]. Data from interview notes and transcripts was synthesized into matrices using the constructed themes (Community, Assets, Preparation, Loss, Displacement, Reconstruction, Health, Veterinary, Status, and Vulnerability), and triangulated with primary and secondary data, consisting of wider literature on the superfloods, livestock and public health and diseases in Sindh [39]. Three second order themes were constructed and interpreted drawing on the ecosocial theory, which aims to improve understandings of epidemiological risk factors, using a multidisciplinary approach to analyse individual- and community-level data in ecological and social contexts [30]. The theory considers biological and environmental factors, but importantly includes political and economic processes, and socio-economic inequalities, and how these express themselves in health outcomes, which is highly relevant in often structurally marginalized displaced populations. The second other themes form the structure of the results section, identifying the multilevel factors, dynamic processes and pathways affecting zoonotic disease risk in displacement contexts [30].

### 2.5 Limitations

The qualitative case study methodology does not aim to provide generalizable research findings, acknowledging that people and phenomena depend on their context and circumstances, although some of its findings may be applicable to similar groups in comparable situations [40].

Selection bias was introduced by selecting only communities which received humanitarian assistance by NRSP in the aftermath of the 2010 superfloods for ethical considerations, while recall bias may have occurred during recollection of disaster and displacement events. The presence of NRSP staff members and a representative from the Sindh Government may have introduced a level of reporting bias.

We present our study findings using the global themes assigned within the thematical analysis. The findings are presented in chronological order along a spatiotemporal scale, starting with a description of the scale of the disaster and loss, displacement experiences and the impact on communities, and return and recovery since the superfloods, and reflect on our findings in the Discussion section.

## 3. Results

### 3.1 The Small Doomsday

**Fig 1:**
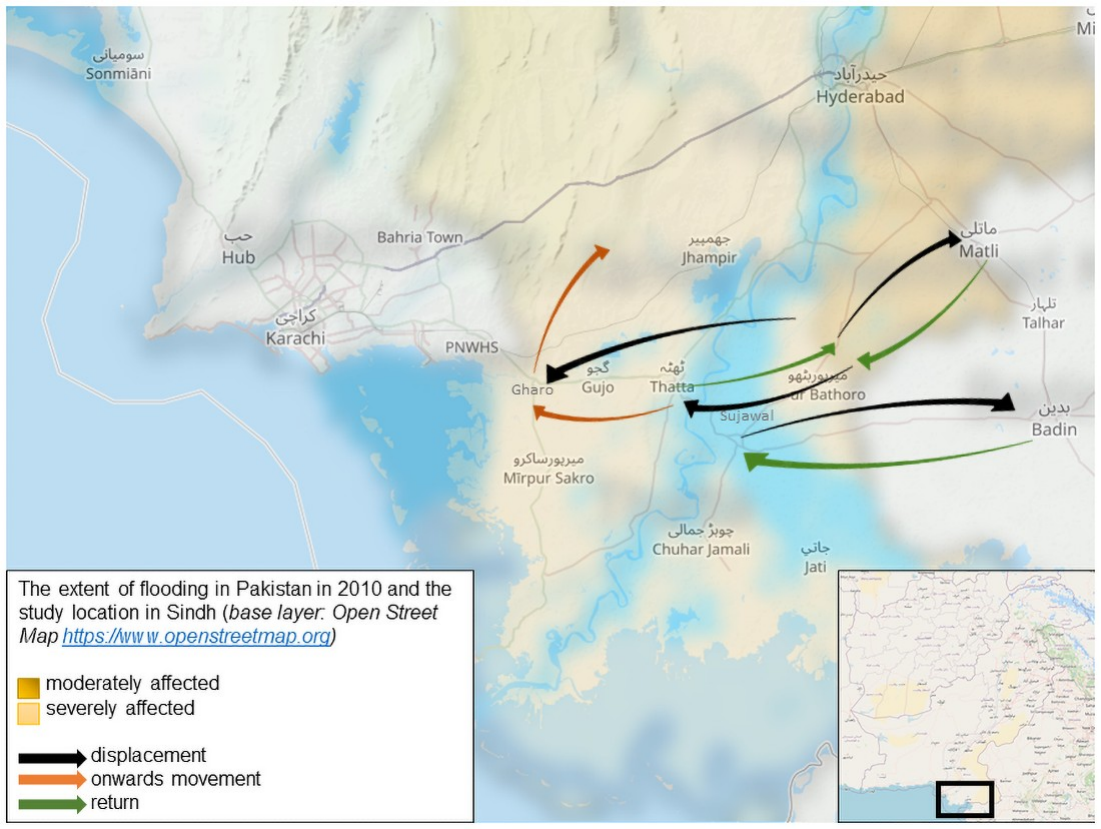
Location of fieldwork in Sindh in Pakistan and major displacement routes during the 2010 superfloods.

Even though the disaster occurred almost ten years before our study, people remember it well: one participant recalls it as ‘the Small Doomsday in our time’ (male livestock owner and daily wage labourer, CAHH2), the most severe disaster they ever experienced. Unstable climatic conditions during the pre-monsoon phase, and heavy rainfall during the monsoon resulted in unprecedented floods in July and August 2010, affecting 20 million people across Pakistan [41]. Floods started in northern Pakistan in July, with the heaviest flooding moving southward towards Sindh in early August. In Sindh, 7.4 million acres of land were flooded and 800,000 houses damaged or destroyed. Infrastructure such as power stations, transmission towers, irrigation systems, barrages, bridges, and roads were lost, many of which have not been rebuilt [42, 43], and 11.7 percent of all health facilities were damaged or destroyed [41, 44]. Over 30 percent of the 7.2 million flood affected people in the province was displaced [41], with 1.3 million sheltering in formal relief camps and many more in informal settlements [45] (Fig 1).

Due to its scale, the disaster was called the ‘superfloods’ in its aftermath, and still referred to as such. The floods affected both harvesting seasons, destroying the *kharif* monsoon crop and hampering planting for the *rabi* spring crops. The province suffered almost half of the total damage to agriculture in the country [41]. Local wheat storage and animal fodder were lost, fresh water supplies were affected, and the Kharif agriculture season aborted, while planting for the Rabi season was delayed due to remaining water, sand and silt deposits [44].

*“On the 5th Ramzan the flood hit*. *The noise of the water sounded like an earthquake*. *There was only one Suzuki [pick up truck] in the village to move the women*, *it was suffocating*. *The men had to walk with the animals*. *There was panic and the bridge was so crowded we were afraid it would break*. *Some people were dropped off in Thatta or Makli*, *but those areas were too crowded*. *It was too far to walk for some animals and these were left behind on the road*. *My brother in law drove in a Suzuki to tell the men herding the animals where the water was*. *When he slept*, *the water came at night and he almost drowned in the car*.*”*

*(female livestock and shop owner*, *CFHH2)*

While all communities were warned about the upcoming floods, either through media, authorities, politicians, police or relatives upstream, only one community collectively relocated before the water reached their village. Unwilling to abandon their livelihoods and important source of nutrition, many people were reported to refuse to relocate without their livestock [46]. Most participants did not physically move until they saw the water streaming into the community ‘with their own eyes’. Houses were rapidly flooded, sometimes inundated as high as six feet, and people scrambled to get away in time.

*“we saw the water coming and had a few days to prepare (…) all houses were flooded up to two-thirds of the doorway (…) wood and straw structures were destroyed”*

*(female livestock keeper and community leader CAHH1)*

In Sindh, over 1.2 million livestock and 6 million poultry were killed [46]. Among our participants, some households lost half of their livestock herd due to drowning. One community lost around 30–40 livestock, another initially had to leave their animals behind to save themselves, returning the next day to see which livestock could still be saved.

*“The loss of animals was more impactful than the loss of our house”*

*(female caretaker of livestock in partnership*, *CEHH2)*.

### 3.2 Livestock over community

In rural Sindh, livestock were described as ‘the centre of the household’ and the most valuable asset, often considered ‘family members’, and often central to the displacement experience. In the chaos of the superfloods, roads were crowded and flood water hampered movement, forcing some people and livestock to walk for four days to safe locations. Animals were lost or killed through road accidents, exhaustion, electrocution, lack of feed and water, while once in the destination location, animals were at further risk of starvation and disease [46]. Those animals that survived the disaster and subsequent displacement, were at increased risk of disease due to exhaustion, starvation, and the mixing with other displaced or host population’s herds. Weak and young animals were most susceptible to diseases, and many were sold cheaply to be able to buy food, shelter materials and fodder.

*“after the floods*, *households in need of money would sell their animal in one of the government allocated livestock markets*, *usually a small ruminant or heifer”*

*(key informant*, *provincial government)*

In some communities local political and/ or army leaders provided buses to move people and sometimes trucks for the animals, however these did not have enough capacity for the entire population. Some households managed to rent cars or tractors for female household members, while male household members walked along with their animals, participants describe how ‘people already weak from fasting had to move’ (male livestock and shop owner, CIHH2).

*“We left the village before the water came; politicians provided vehicles [to evacuate] the people*, *while animals walked”*

*(male livestock owner and daily wage labourer*, *CGHH1)*

Respondents suggested that displacement often occurred in stages, as some initial destination areas were subsequently flooded or considered unsuitable living environments, eventually displacing some of our participants up to 50 kilometres from their homes. Communities supported by the army were housed in formal relief camps, without space provided for their animals, causing households to split up as male family members stayed with the animals. Women remained in formal relief camps with young children, as the traditional caretakers in a household, with one female participant commenting: ‘first the children get sick, then us’ (female livestock owner, CHHH1).

*“we were relocated by buses from the government (…) we could choose to go to Makli*, *but we went to Gora to stay close to our animals (which was brought by the) men and boys by foot (…)*.*”*

*(female livestock keeper and community leader*, *CAHH1)*

In other locations where it took time for formal assistance to arrive, livestock keepers moved on to find a suitable location with fodder and water for their animals, while the poorest households without livestock stayed put, dividing communities. A participant from one of the poorest households reflected: ‘in times of disaster it is every man for himself’ (female caretaker of livestock in partnership, CAHH3). Throughout the country, a third of displaced moved at least twice [42].

*“people prioritize their animals*, *[for instance] during the 2010 superfloods people insisted that their animals were rescued first (and displacement) destinations are often chosen based on their suitability for animals*.*”*

*(NRSP)*

During displacement, animals suffered from suffocation due to what was described as a ‘swelling of the throat and nose’, fever, and bloat. In the dry, hilly areas, displaced people found that Foot and Mouth Disease (FMD) was (and remains) endemic. Participants used traditional treatment methods before calling a veterinarian in late stages of disease, which spread easily with animals herded closely together during displacement. Some participants moved with their animals away from crowded settlements, partly because of increased disease risk from ‘other communities’. This distrust of others was reflected by one participant who mentioned that ’outsiders bring bad hygiene into the village’.

*“In 2010 we [first] moved to the dams next to the river (…) the Shirazi leadership moved all people to Gharo (…) where there was FMD and we did not want to live too close to other people because of these diseases [so the community split up]”*

*(male livestock owner and daily wage labourer*, *CAHH2)*

*“In Makli*, *lots of animals were kept in one place so diseases spread [we] lost 4 buffalo*, *5 cattle and many goats*, *[the boys] moved to another hilly area with the animals”*

*(male livestock keeper and school teacher*, *CEHH1)*

While none of the participants directly acknowledged the possibility of disease transmission from livestock, we found that many were indeed aware of zoonotic disease risk, as the increase in respiratory and gastrointestinal infections during displacement was at least partially attributed by respondents to a lack of hygiene living close to their livestock. Although most households would usually not sell sick animals or use their produce, many were forced to during displacement. While milk was not always boiled, respondents mentioned this is as much a result of a lack of time, fuel, and alternative foods, as a lack of awareness [47], with several respondents referring to relevant cultural and disease control practices: ’milk is boiled for preservation [and] to kill the germs’ (female livestock and shop owner, CFHH2). Hands are traditionally washed with water or sand if the former is not available, before and after handling animals.

Participants reported that the burden of infectious diseases increased during disaster displacement, including fever, respiratory and gastrointestinal disease, which in the literature is primarily attributed to a lack of safe drinking water and sanitation facilities [48], while scorpion and snakebites caused fatalities. While the humanitarian health cluster was providing assistance in the region through UN agencies, international and national NGOs, and vaccinations were provided in formal camps, none of our participants mentioned receiving free healthcare. Instead people visited and paid for overburdened private health facilities near their respective destination areas. During and after the superfloods, FAO provided limited livestock vaccinations and feed, however this was not coordinated by national disaster management authorities. Even current seasonal FMD vaccination campaigns only reach about 10 percent of formally registered cattle, according to a local veterinarian. None of our participants reported receiving any livestock support and gaps in veterinary disease surveillance resulted in inconsistent zoonotic disease prevalence data collection (DoL).

*“during the 2010 superfloods*, *many people were displaced to Hyderabad and Karachi*, *others moved to the roadside (…) organisations provided livestock vaccinations*, *medication*, *the government provided shelter*, *food was provided but no livestock feed*.*”*

*(provincial government official)*

*“in 2010 assistance was provided in camps and along roads and river banks*, *but because veterinary clinics were flooded and staff evacuated*, *the response was slow; while medication and vaccines were supplied to camps*, *diseases got worse”*

*(Department of Livestock Thatta)*

Humanitarian assistance hardly ever reached those herding the livestock in remote areas, while those participants who moved east rather than west, away from the Indus and further from Karachi, were subsequently cut off from formal relief efforts by flooded roads, and became dependent on host populations. As many communities and households split up, rather than remaining a mutually supportive collective, displaced households became dependent on new community structures as outsiders [22]. This division of households had great psychosocial effects, with one of our female participants describing the experience as ‘mental torture’ (female livestock and shop owner, CIHH1). Another female participant mentioned feeling ‘like an orphan’, away from her husband (female caretaker of livestock in partnership, CAHH3). Others felt uncomfortable between unknown households from different tribes and returned to dams or bridges closer to home, sometimes even returning while their village was still flooded.

### 3.3 Relief and recovery

Respondents mentioned returning to their villages after 2 to 4 months, borrowing or renting vehicles for transport, sometimes paid for by selling more livestock. Some villages were still flooded 4 months following the disaster, and only those houses built on higher ground could be used. Faced with destroyed houses and agriculture, people remained dependent on external assistance. Humanitarian and government agencies provided shelter material, tents, handpumps and food, prioritizing the poorest households. A restocking initiative resulted in poor households selling the provided goats, as they were in need of cash and unable to feed the animals. Some participants found their houses looted, and stolen IDs meant they were not eligible for electronic cash assistance cards [41]. While receiving humanitarian assistance within the relief camps, on return to their home village women were less likely than men to have an identity card, hampering access to assistance in the aftermath of the superfloods [45].

The National Disaster Management Authority (NDMA), which had only been recently established at the time, formally coordinated the initial humanitarian response, supported by the army, Provincial Disaster Management Authorities (PDMA) and District Disaster Management Authorities (DDMA), the latter headed by the District Commissioner, a government appointee [49]. As local governance structures were still in transition following the 18th amendment to the Constitution, whereby power was devolved to the provinces, including the responsibility for health and livestock, the PDMA and DDMA had limited response capacity and structures [46, 50]. Due to the scale of the disaster, national and international aid agencies and charities supported the provision of emergency assistance, coordinated through the humanitarian cluster approach, handing over delivery of services to ‘early recovery working groups’ by April 2011 [41, 48]. As the disaster took place during Ramzan, the ninth month of the Islamic calendar observed as a month of fasting, prayer, reflection and community, private philanthropical contributions were significant. However due to the lack of disaster response infrastructure and capacity, the response in Sindh remained relatively slow compared to the rest of the country [41].

*“Most assistance was provided close to Karachi*, *which was easier to reach for people with private gifts”*

*(NRSP Sujawal)*

Having survived both disaster and displacement, in most communities the health of humans and animals was primarily affected following their return. Nationwide, around 77 percent of people reported illness within their household in the six months following the floods [42]. Data from the health cluster showed that within the first year over 37 million medical consultations were reported in flood affected districts, the most common being acute respiratory infection (23%), skin diseases (11%), acute diarrhoea (9%) and suspected malaria (6%) [41], a common disease which is usually prevented by the use of mosquito nets, which were not available during displacement. Respiratory and gastrointestinal infections in many cases worsened after returning to polluted water sources in home villages, and by October 2010 laboratory testing confirmed the outbreak of cholera [51]. Water from handpump wells was contaminated, causing disease and the death of four children in one village.

*“I suffered from Hepatitis C after the floods*, *it was common and some people died because of it*. *After the floods*, *the handpump water was tested by NGOs [and identified] as a cause for diseases”*

*(male livestock keeper and school teacher*, *CEHH1)*

At the time of research, sanitation and water supply in the villages remain limited. The poorest households do not have latrines, whereas the less poor families in larger villages have only recently started constructing their own. Water sources vary from handpumps, usually shared among households, to irrigation canals and surface water, in which we often noticed bathing buffaloes. While the superfloods polluted the few water sources within communities, the provision and quality of water has always been, and remains, poor. Across rural Sindh the rural population lacks access to water supplies, while most of the rest not suitable for consumption [52]. Almost every village visited had an ongoing drainage problem in agricultural fields since the floods, for which they blame the lack of governmental response.

*“there is enough water in the Indus*, *but the water management system is bad”*

*(male livestock kowner and migrant worker*, *CHHH1)*

Those households with savings left after the disaster and displacement, pooled funding to rebuild brick houses. As we visited the communities nine years after the superfloods, we noticed that most participants still live in small 1–2 room *kutcha* houses built out of wood and mud, while less poor households constructed brick homes. The largest house we visited–owned by the community leader—consisted of four rooms around a central covered space used for storage and parking. One household spent three years reconstructing their brick home using a bank loan, which was repaid by selling buffaloes. Some households managed to replace *kutcha* houses with brick, while a few individuals managed to increase their livestock herd. The households interviewed now owned up to 24 livestock, usually a combination of buffalo, cattle and goats. The poorest households used a ‘partnership’ system, whereby livestock is not owned by the caretaker themselves, but on loan from a relative or wealthier acquaintance. All produce is kept and used by the caretaker, while profit on sales is returned to the owner.

*“Before the 2010 floods we had 30 buffalo and cattle (…) some died*, *some were affected by disease and were sold*, *at returning to the village we had 20 animals left”*

*(male livestock owner and daily wage labourer*, *CAHH2)*

The main livelihood of the communities remains subsistence agriculture and livestock keeping, with animals central to people’s lives and livelihood, for both economic and cultural reasons [53]. Cattle and buffalo are kept for milk, and surplus is sold on the market, while goats are kept for their meat or sold for cash, to buy agricultural input, food, or pay for services such as veterinary and healthcare. Both men and women milk the animals, depending on household traditions, and some poorer households milk other people’s cattle. Some households own a few acres of agricultural land and/ or work as daily labourers for other smallholders or a landlord out of necessity [33].

*“(…) the tenancy act is not implemented*, *there remain issues with land boundaries*, *water management and cost sharing*. *In Sujawal farmers share at 25/75 basis (…) 1–2 acres of land is not enough to live from*, *therefore people also have to conduct daily wage labour.”*

*(FAO)*

Participants mentioned it took between 3 to 6 years for agriculture production to return to pre-flood levels and villages to be reconstructed, with support from national and international governmental agencies and civil society organisations. Girls’ access to education improved after the influx of aid, with larger communities now providing education to young children (up to 10–12 years), although there are concerns about the quality. While most villages are now connected to the electricity grid, power is only provided between 2–8 hours a day. For many however, life has become harder since harvests and livestock were lost, and the prolonged inundation of fields and water sources caused permanent salinization.

*“[during the superfloods] all harvest was lost and it took three years to recover (…) the floods affected the supply of fresh drinking water*, *now they have to go as deep as 50 feet to find it*. *(…) while they are now in a ‘better’ position*, *we are still worse off than before the floods*, *as there is no daily wage labour*.*”*

*(male livestock keeper and school teacher*, *CEHH1)*

Income levels and purchasing power among participants therefore remain low, directly affecting food intake. To contribute to household income, young male household members often work as daily wage labourers in factories or doing road construction, while others migrate to urban areas, in particular after harvest season when there is no local agricultural labour. Women contribute to household income through selling embroidery. All communities had small shops, selling basic items such as soap and snacks, with some selling basic medication. Transportation options were limited, while some households owned motorbikes, ownership of chin-chi’s (three-wheeled motorbike/car hybrids) and cars was very rare. Small trucks (‘Suzuki’) were hired to transport goods and people to and from larger towns or cities for access to markets and services.

Recently, poverty and prices increased, with participants reporting a decrease in purchasing power due to ongoing inflation and the lack of daily wage labour, partially an unintended result of the recent closure of factories due to political counter-corruption measures. Interestingly, the poorest households mentioned little change, or being ‘better off’ than in 2010, as they had no livestock at that time, and they did not have to repay animals held in ‘partnership’ lost in the superfloods. Furthermore, with increased funding and visibility, NGOs and relief agencies increased their involvement after 2010, and in some communities the position of girls and women improved slightly by facilitating education and paid employment for women. Among others, NRSP has trained communities in disaster management and formed disaster committees with disaster preparedness plans, which include safe areas identified together with the communities suitable for livestock. In 2015 this system was used with line departments including PDMA, health and livestock, and resulted in fewer losses.

## 4. Discussion

The ecosocial theory of societal patterns of disease distribution posits that in socially unequal societies the absolute burden of disease rests on those with less power and fewer resources [27], as it increases the risk of excess exposure to hazards and pathogens, inadequate healthcare, ecosystem degradation and lack of land ownership, resilience and agency [29]. These vulnerabilities are largely constructed, with people made vulnerable through political and social inequalities. Even before the superfloods, Sindh province could be considered disadvantaged, with one of the highest rates of inequality and lowest literacy rates [54], and the highest levels of malnutrition and food insecurity within Pakistan, with chronic malnourishment affecting the immunity of 84 percent of the population [44]. Inequality, economic marginalisation and the lack of a comprehensive healthcare system has led to high levels of infectious diseases, including zoonoses, although these are rarely formally diagnosed among disadvantaged population groups in rural Sindh [55, 56].

The superfloods disaster exacerbated these chronic vulnerabilities [57]. Disaster displacement only occurs when people are vulnerable to natural hazards, with those lacking resources living in low-lying riverine areas most at risk [46]. Mustafa describes how vulnerability to floods in Pakistan depends on land ownership, sources of livelihood, political and institutional connections, class and gender, thereby affecting individuals within communities differently [58]. Historical developments resulted in a democracy heavily influenced by feudalism, and high socio-economic inequality in rural Sindh [59], exacerbating the impact of hazards by the unequal distribution of resources, reserves and ability to prepare. The British colonial administration institutionalized the Mughal feudal practice of landownership and tax collection by agents by granting land owners property rights to secure their revenue base and political interests [33, 34, 54], while tenants worked the land, paying for cultivation themselves, often through loans either from the landlords or money lenders [33]. Currently, almost 64 percent of rural families are landless tenants, often indebted to a landlord for generations [44]. Agriculture land remained privatized after independence, with landlords continuing to exercise political control, irrigation and water distribution [54], affecting all of our participants’ communities. This persistent system has caused Sindh to have the largest rural-urban social gap in Pakistan, according to the World Bank [54].

Structural inequality before and during displacement was reflected in the marginal geographic location of settlements, stress and trauma, the availability of sufficient quantity and quality food and water, and structural lack of access to adequate health, veterinary and other services [45]. Our study indicates that available connections, social status and resources before disaster played a significant role in determining people’s disaster displacement experience. Better connected communities received early warnings, and transportation support from government, tribal affiliates, or political allies—usually the landowners on whose land the community lives and works. Limited availability and capacity of transportation during the superfloods resulted in a divide between classes within communities however, whereby the poorest households were left to their own devices.

Communities, households and individuals therefore experienced displacement differently, depending on available resources, the position of the community within a wider (tribal and political) relations network, the position of the household within the community, and the role of individual household members. These determined whether they were able to afford or receive transportation and/ or whether were hosted in a destination location conducive to livestock, with some of the population groups considered most vulnerable by humanitarian actors ending up away from animals, particularly affecting women. Structurally disadvantaged in decision making, resource allocation and access to education and health services, women are less likely to access healthcare, which Shaikh and Hatcher (2005) attribute to their limited role as decision maker and control over resources, limitations to travelling alone, and likelihood of facing language barriers due to inequal education [60].

Movement, separation and uncertainty caused exhaustion, stress and other psychosocial issues with a detrimental effect on immunity, including in animals. Despite these and pre-existing vulnerabilities to disease, during displacement zoonotic disease dynamics were complex and non-linear. Livestock were central to people’s decision making during the superfloods linked to transport options and destination location. The latter was often determined by livestock owners based on their knowledge of infectious human and animal disease transmission risk. While key informants claimed that rural communities were generally not aware of zoonoses, risk factors and routes of transmission [47, 61], our study showed a more nuanced picture. Zoonotic health conditions in the communities were already challenging before the disaster, with most respondents lacking resources for veterinary health care, while the misuse of medication and unregistered quacks when resources are available, increases the risk of infectious disease and antimicrobial resistance (AMR) [55]. Veterinary and public healthcare in Pakistan are characterized by a mixed system of public and private providers, with many public sector employees working in private practice after hours, while public services are underused due to a lack of trust and general dissatisfaction [25, 55, 60]. These studies report a generalised believe that ‘free medicine is bad medicine’, an attitude that the authors believe is rooted in colonialism, with outbreaks such as the recent HIV epidemic in children in Sindh, caused by the reuse of needles by someone posing as a medical professional, exacerbating this mistrust [62]. Poor governance and low public investment, partially caused by structural adjustment programs enforced by international donors through the International Monetary Fund (IMF), have resulted in chronically under-resourced public services [25, 61, 63, 64]. During the superfloods, public health systems and health facilities in destination areas therefore quickly became overburdened once displaced sought treatment.

This shows that structural and chronic drivers, rooted in historical and political structures, affected people’s vulnerability to disaster, displacement and zoonotic disease. The availability of informal networks and resources at origin, transit and destination locations, and the availability and prioritization of (humanitarian) assistance, determined stress, food, water and sanitation. This was further complicated by unhygienic living conditions in displacement, which increased susceptibility to disease. Ultimately, people’s ability to respond to these disease risks was primarily driven by their pre-existing status, socio-political connections and resources.

Rather than framing zoonotic disease risk in displacement as a result of increased pathogen interaction between animals and humans, such ‘risk’ needs to be reconceptualized as the interaction of biological, environmental, socio-economic and political factors. Movement itself is not a determinant, rather the process which alters biological susceptibility, based on pre-existing vulnerabilities grounded in structural inequalities, playing out differently dependent on resources and connections during displacement. Considering these complexities, the ecosocial model seems an appropriate fit based on the experiences garnered from our respondents.

Social science research adds crucial insight into complex local, social and political processes affecting disease transmission and control. Zoonotic disease risk in displacement contexts cannot be reduced to one narrative, and must be considered with an interdisciplinary approach to uncover intersections between biomedical, historical, political, and behavioural factors. With this study, our aim was to provide depth and context to a so far under-explored topic, rather than provide data for extrapolation, however our study does provide contextualised data and theoretical concepts useful for subsequent qualitative and quantitative study to further engage with the topic [65].

## 5. Conclusion

The findings from our study speak to several areas where mitigation of infectious diseases and the effectiveness of support services generally during displacement can be greatly improved. These areas of improvement include targets before, during, and after displacement. The findings from our study make it clear that the bulk of disease prevention, including the prevention of zoonotic spillover, occurs before the disaster that necessitates displacement transpires. In order for zoonotic disease mitigation activities to be most effective, it is necessary to engage with and address vulnerabilities within the population ahead of time.

In order to improve zoonotic disease mitigation activities and the general wellbeing of the displaced communities after the event has occurred, responders and donor agencies need to acknowledge and better accommodate livestock in their responses, as livestock showed to be an important factor during the 2010 superfloods’ disaster displacement of rural communities. The findings from our study make it is clear that this will not only increase the effectiveness of existing interventions and help families stay together during displacement but also leave them better able to resettle and re-establish their livelihoods post-displacement. From our study and previous work in this area, it is apparent that it was upon returning to their communities after having been displaced when risk of disease outbreaks was the greatest. Increased support during this crucial stage of resettlement is therefore warranted. These findings should support enhanced understanding of zoonotic disease dynamics during displacement, providing entry points for inclusive discussions for better interventions and policies. By involving communities in these processes, their agency can be increased to impact the division of power, land and resources. Addressing pre-existing inequality and vulnerability will improve preparedness, adaptation, resilience and response to disasters and displacement, thus reducing zoonotic disease transmission risk.

Finally, a key finding from our study was the heterogeneity of experiences and vulnerability within the displaced communities. The markedly varied trajectories and needs of different subsets of the community need to be taken account in order for interventions and aid to be delivered effectively. This is an area where the design of interventions and services would benefit from greater input from other disciplines, including anthropology. This will require donor funding and political will, as well as engagement of both international and national stakeholders for more inclusive, interdisciplinary research and response.

## Supporting information

S1 File Research tools. (DOCX)
