# Supplementary material for: Disaster displacement and zoonotic disease dynamics: The impact of structural and chronic drivers in Sindh, Pakistan
Source: PLOS Glob Public Health. 2021 Dec 8;1(12):e0000068. doi: 10.1371/journal.pgph.0000068 (PMC10021430; doi:10.1371/journal.pgph.0000068)
Supplement: S1 File — (DOCX) [file pgph.0000068.s001.docx]

**INTERVIEW PROVINCIAL LIVESTOCK/ HEALTH AND DISASTER MANAGEMENT EXPERTS**

Objective: to gain the (livestock, health and disaster management) expert’s perspective of zoonotic disease risk in displaced animals and humans. To determine which communities can be accessed and interviewed. These interviews will be conducted with authorities in provincial (Karachi, Hyderabad) offices.

**1. PERSONAL INFORMATION**

1. What is your current role and background?
   1. Prompt: activities on a regular day vs during emergency such as disaster/ disease outbreak; availability records (SWOT)
   2. Probe: achievements (what are you most proud of); priorities, how do you prioritize?
   3. Probe: who are your main counterparts?
2. To what extent does your current role cover zoonotic diseases OR displacement?
   1. Disease outbreaks; chronic/ endemic disease
3. Which policies do you follow for prevention, response? [share (livestock) policies]
   1. Probe: who was involved in the development, national government, local/ village community/ individuals making a difference; your role?
   2. Probe: issues for improvement

**2. SINDH GENERAL**

1. Can you describe the situation in Sindh?
   1. Prompt: livestock keeping, health, services, cultural, poverty/ land issues
   2. Probe: have these changed in the past (10) years
   3. Probe: what are your main concerns (current drought?)
2. Can you describe a rural household in Sindh?
   1. Prompt: socio-economic (land ownership), housing, education, household roles/ responsibilities
   2. Probe: what livestock, how is livestock kept, changes in livestock management, use of products/ marketed
3. What services/ capacities are available?
   1. Prompt: Laboratory, medication/ vaccination
   2. Probe: When are these used, who decides?

**3. PREVENTION AND RESPONSE**

1. How healthy are the rural households would you say?
   1. Prompt: adults/children; health, endemic
   2. Probe: are there areas where people/ animals are healthier? Extent of zoonotic diseases/ change in patterns?
2. How do you prevent zoonoses?
   1. Prompt: policies, surveillance, response? Disease control compartments? Laboratory confirmation? Vaccination strategy/ campaigns?
   2. Use of media, schools?
   3. Probe: how does the policy affect zoonotic disease transmission and why?
3. Risk management in relation to zoonotic diseases? How do you prevent zoonoses?
   1. Prompt: policies, surveillance, response? Strategy? Education of rural households/funding for initiatives/ information for farmers/ advisors visiting households/ or working through vets/doctors?
4. How do you / your organization respond to: zoonoses/ (current) drought/ displacement?
   1. Prompt: influence, funding, education, training, how implemented (by whom, how)
   2. Probe: what was the reason to choose these interventions?
   3. Probe: what was good/did anything go wrong?
   4. Prompt: could it have been prevented?
   5. Probe: reporting (notifiable disease?)
5. To what extent does your work/department get involved in prevention of disasters?
   1. Prompt: natural disasters leading to displacement/ disasters linked to spread of disease; how, improvement?
   2. Probe: have you had any health issues relating to livestock disease crossing over from animals to the human population?
   3. Probe: Is there coordination with other stakeholders? Who is ultimately responsible?

**4. VULNERABILITY AND RISK**

1. What do you think puts people at risk of zoonotic diseases?
   1. Prompt: what are main zoonotic diseases that you have had to deal with? Names.
   2. Probe: causes/ risk factors/(political, environmental, economic, etc) /
2. How much does displacement affect the risk?
   1. Prompt: (political, environmental, economic, etc) risk factors/ displacement
   2. Probe: Example of last displacement (perceptions, assumptions), actions of communities
3. What is the current status of [health/ livestock/ displacement]? How healthy are rural households?
   1. Prompt: are disaster displaced more at risk of zoonotic diseases
   2. Probe: locations, main concerns
4. Where do you think these diseases originate/ what are the causes? Example
   1. Prompt: Authorities (policies), experts (response, services), farmers (local risk factors)
   2. Probe: what services are available
5. Do you think landlords/ communities have a role in (preventing) zoonotic disease transmission? Example
   1. How do you work together with landlords on this issue?
   2. Prompt: (lessons learnt about) risk factors: poverty, tradition, culture, responsibility
   3. Probe: how much access to communities do you have to prevent / ensure resilience; through whom?
6. Is there anything that you feel I should have asked you or that you want to tell me in relation to this issue?

With many thanks for your help

**INTERVIEWS: EXPERT INTERVIEWS LOCAL LIVESTOCK / HEALTH SPECIALISTS**

Objective: to gain the expert’s perspective on zoonotic disease risks within the displaced community, including prevalence of zoonotic diseases in humans and animals.

**1. PERSONAL INFORMATION**

1. What is your current role and background?
   1. Prompt: qualification, describe activities on a regular day, during emergency such as disaster/ disease outbreak [current drought]
   2. Achievements: what are you most proud of?
   3. Probe: priorities, concerns
2. Describe the community (-ies) you serve
   1. Prompt: origin, type/ quantity livestock, tradition/ cultural, socio-economic, spending patterns; change in the last (10) year
3. How healthy is the community?
   1. Prompt: information levels about health/diseases, most worrying, prevalence, reasons?
   2. Probe: where does treatment take place (home visits, clinic?)
   3. Prompt: barriers for displaced: language, documentation, fear, cost
   4. Probe: registration; reporting/ reports (SWOT)

**2. PREVENTION AND RESPONSE**

1. How do you prevent zoonoses?
   1. Prompt: policies, surveillance, response? Disease control compartments? Laboratory confirmation? Vaccination strategy/ campaigns?
   2. Use of media, schools?
   3. Probe: how does the policy affect zoonotic disease transmission and why?
2. How do you / institution respond to: zoonoses/ displacement?
   1. Prompt: protocol, influence, funding, education, training, how implemented (by whom, how)
   2. Probe: how did you choose these interventions? Example? [intention versus practice]
   3. Probe: what was good/did anything go wrong? could it have been prevented?
   4. Probe: who are your main counterparts? How do you connect with authorities?
   5. Do you use media to inform the public? Which, how
3. Are there any laboratory services, medication available?
   1. Prompt: where, expense?
   2. Probe: when do you report disease (notifiable disease?)

**3. VULNERABILITY AND RISK**

1. How do people become at risk of zoonotic diseases?
   1. Prompt: (political, environmental, economic, lifestyle, slaughter, cooking, etc) risk factors/ displacement; is there proof/ data?
   2. Probe: Example of last displacement (perceptions, assumptions), actions of communities
2. What is the current status of [health/ livestock/ displacement] in this area?
   1. Prompt: disaster, prevalent / endemic disease, zoonotic diseases
   2. Probe: locations, main concerns
   3. Are there areas where humans/ animals are healthier, why?
3. Where do you think these diseases originate/ responsibility?
   1. Prompt: Authorities (policies), experts (response, services), farmers (local risk factors)
   2. Probe: what services are available
4. Do you think the authorities/ landlords/ communities play a role in zoonotic disease transmission/ prevention? Example
   1. Prompt: policies, risk factors: poverty, tradition, culture
   2. Probe: how can this be improved
   3. Probe: how much access to communities do you have to prevent / ensure resilience; through whom?
5. Can you give me an example of a case where you think people who have moved here got infected/ transmitted zoonoses from livestock and how it was dealt with?
   1. Probe: what was good/did anything go wrong?
   2. Prompt: could it have been prevented?
   3. Prompt: local/ indigenous beliefs; customs, traditions
   4. Probe: how are these applied? Protocols, (cultural) behavior?
6. Is there anything that you feel I should have asked you or that you want to tell me in relation to this issue?

With many thanks for your help

**INTERVIEWS: COMMUNITY LEADER**

Objective: to collect information about the households in the community and themes such as poverty, housing and humanitarian assistance.

**1. GENERAL INFORMATION**

1. Can you describe the community?
   1. Prompt: when established (name?); organization village/compound; number of families; education/ facilities; transportation (to where, for what?)
   2. Prompt: tribe, caste, leadership, community roles (tradesman, herder), lineage, marriage, basic unit: household composition
   3. Probe: has the community changed (size/ households) during displacement?
2. What (type/ quantity) livestock is kept by local households?
   1. Prompt: origin, type/ quantity livestock, ownership
   2. Probe: were these livestock owned before displacement? Changes/ why?
   3. Who looks after them/ gender/ how/ where?
3. Can you describe the drought event?
   1. How is the response now different from normal dry times?
   2. Why? Prompt: agricultural practices, kind of feed, water
4. How would you describe how displacement happened here and what effect it has had on people and their lives.
   1. Prompt: Why this location (push/ pull), would you have chosen another location if not a)
   2. Did you move with the entire family? If no where / what do others do?
   3. Can you describe what happened to you and your herd and how it affected your life?
   4. Probe: what animals are used to move? travel measured by daily span, 8-9 miles?
5. Have you been displaced before?
   1. Please describe (as above)
   2. Why do you think it is different this time?

**2. VULNERABILITY AND RISK**

1. What do you think puts people at risk of diseases?
   1. Prompt: which diseases, why (political, environmental, economic, lifestyle, cooking, etc)
   2. Role of livestock in these diseases (case/ slaughter/ preparation)
   3. Probe: Example of last displacement (perceptions, assumptions), actions of communities
2. How much to you think displacement has affected the risk/prevalence of disease?
   1. Probe: has it affected the livestock husbandry? Has that affected humans getting diseases from livestock?
   2. Probe: how much are people worried about these sorts of diseases that might have come from livestock? What precautions might they take? Do they work in your view?
3. Where do you see the responsibility lies for preventing these diseases?
   1. Prompt: Authorities (policies), experts (response, services), farmers (local risk factors)
   2. Probe: what services are available

**3. OCCURRENCE AND PREVENTION**

1. How do you prevent disease?
   1. Is there an area where animals are healthier?
   2. Which animal is associated with good health?
2. Can you tell me about community responses to: zoonoses/ displacement?
   1. Probe: protocol (isolation, restrictions, who cares for?); what was the reason?
   2. Probe: reporting, contact with authorities? Working with households to change their (hygiene) practices?
3. What kind of assistance has the community received from outsiders? Example?
   1. Prompt: type, quantity
   2. Probe: who received what, why?
   3. Probe: are you happy with the assistance provided?
4. I am keen to speak to households in the community that have displaced with/ without livestock, could you identify these and introduce me?
5. Is there anything that you feel I should have asked you or that you want to tell me in relation to this issue?

**INTERVIEWS: HEAD OF HOUSEHOLD (if other than head of community/ herder)**

Objective: to learn about the displacement experience, livestock history, physical resources in household, problems of farming/ risks of health, disease, to determine how vulnerabilities to zoonotic disease transmission risk has changed during displacement.

**1. PERSONAL INFORMATION**

1. Can you describe your household?
   1. Prompt: basic unit/ household composition, roles (daily routine, activities, marketing, shopping)
   2. Prompt: children/ ages; education/ schools (location, level, who attends, why (not); (other) jobs; disability
   3. Probe: emigration, do you have any household/ family/ clan members outside of the community, where are they?
2. Can you tell me/ show me how you live?
   1. Prompt: shelter (#rooms), sheds, hygiene, availability water and food (+source), toilet, bathroom, soap, cooking facilities, electricity, fuel, internet/ phone connection, mosquito net
   2. Probe: how do you build your shelter/ shed, why [perceived risk decrease?]
   3. Probe: can you take me through your daily life
3. Do your children go to school? Did they go to school previously? What kind/ type of school?
   1. How often do your children miss a school day because of health?
4. Do you have enough to eat? What do you eat? Where does it come from? How do you prepare it?
   1. Do you have more or less food now following displacement?
5. Do you own any livestock?
   1. Prompt: origin, type/ quantity livestock; who takes care of these?
   2. Probe: did you own any before displacement
6. What do you do with the animals?
   1. Prompt: transportation, food, bride payment, Eid, offering
7. Household income (other)
   1. Prompt: produce, remittances
   2. Spending: decision making, highest (wedding etc) > lowest
   3. Is there some kind of insurance system? Pooled funds (especially in regular disaster affected communities)

**2. DISPLACEMENT**

1. Can you describe the drought (or flood) event?
   1. How did you respond: change agricultural practices, move grazing, water?
   2. What do you do during normal dry times and how/ why was it different this time?
2. Where did you live previously?
   1. Probe: how did you choose this destination, who made the decision?
3. Can you describe your (displacement) experiences and challenges?
   1. Probe: what animals used to move? travel measured by daily span (8-9 miles)?
   2. Prompt: choice of destination, transport, family members, assets (animals), main issues, insurance
   3. Probe: have you been displaced before? can you describe that experience and whether it was similar/ difference? Return?
   4. Probe: How do you share information about previous experiences? Journal keeping?
4. How has the displacement affected you and your family?
   1. Prompt: resources, environment, shelter, nutrition, income generation of livestock vs non-livestock income sources before and after displacement, spending priorities, animal handling, grazing, slaughter, health
   2. Probe: How do you address these changes? Examples.
5. Can you tell me about your animals and how their situation changed due to the displacement?
   1. Prompt: which animals/ who looks after them (gender) , since when, why these types of animals, shelter (depend on species?), use of animals (changes?), health
   2. Probe how their situation changed due to the displacement/ Did you lose livestock? When and how? How many? death, sales, slaughtered, other?
   3. Probe: have you changed the way you look after them? How – describe husbandry before and now
   4. How did all these things impact your life?

**3. VULNERABILITY AND RISK**

1. How are you feeling today? Do you have any complaints?
   1. How often do you feel like that?
   2. Is there a difference before and after displacement? Concerns, worries?
   3. Did it stop you from working/ school?
2. Can you give me an example of the last time you/ your family member caught a disease?
   1. What caused it? Did you change anything because of this?
   2. What assistance did you seek/ ask for, did you receive it? Relationship to NGOs?
3. When you are ill, do you seek health treatment?
   1. Why (not): location, expense (example)
   2. When do you decide to seek healthcare? Role religion, previous experience, word of mouth, funds (threshold)
   3. Probe: Which specific facilities? [compare with local health data?]
4. Do you use medication, from what source, how much do you spend on these? What resources do you use to pay for treatment/ medication?
   1. Prompt: Income, loan, remittances?
   2. What do you do with any leftover medication? Do you ever use other people's medication?
   3. Do you have any medical kit in the house?
5. Do you or your family members suffer from diarrhea at the moment? How often does this occur? What do you believe causes it? What do you do about it? Did you have these complaints before displacement as well?
   1. Prompt: health/ treatment; medication
6. What do you think puts people at risk of diseases?
   1. Prompt: (political, environmental, economic, lifestyle, slaughter, cooking, etc) risk factors [animals: why do you think that?]
   2. Probe: Example of last displacement (perceptions, assumptions), actions of community

**ANIMAL HEALTH**

1. Which of your animals is most healthy?
   1. Which products are most healthy for your/ families health? Why? What do you do with them/ how do you prepare these?
   2. Is there an animal which poses a risk to your or your children's health?
   3. Have you or family members ever been bitten by a dog? How often did this happen? What did you do when it happened?
   4. Prompt: health/ treatment; medication
2. Can you describe animal illnesses and how you would normally deal with those:
   1. Prompt: severity, symptoms (example)
   2. Probe: Why do you think your animals are more/ less diseased (causes)?

Probe: Help in the community from other households?

1. Prompt: deal with it yourself – Change what you do? how? Separate animals from humans? Split herds? [map]
2. What else do you do with your animals if/ after they are sick (example last time an animal was sick).
   1. Prompt: vaccination, veterinary (or why not?); and if that does not help?
   2. Is there an area where / time when animals are healthier? Why do you think this is?
   3. Probe: Do you report on disease?
   4. Probe: Where do you get your information? How do you know what to do?

NB: If no animals: where are they now and what could have been done so you could keep the animals?

**4. SERVICES**

1. Have you received any [humanitarian] support/ assistance?
   1. Prompt: type, from whom, how do you receive it, is it sufficient/ happy?
   2. Probe: Can you tell me what other services you access and how?

**5. FUTURE**

1. Can you tell me what your plans are?
   1. Prompt: stay here, return, move on?
   2. Probe: what would influence decision?
   3. Prompt: Policies, assistance, services [prevent protracted displacement]
2. What is your main concern in relation to the health in your family? Why is this so important to you?
   1. Probe: what have you done about it?
3. Would it be possible to speak to the household members responsible for herding/ taking care of the livestock?
4. Is there anything that you feel I should have asked you or that you want to tell me in relation to this issue?
5. Are there any issues the interview brought up you would like more guidance on?

With many thanks for your help

**INTERVIEWS: LIVESTOCK HANDLER AND HERDER**

Objective: to learn about their displacement experience, livestock history, physical resources in household, problems of farming/ risks of health, disease etc, to determine how vulnerabilities to zoonotic disease transmission risk has changed during displacement.

**1. PERSONAL INFORMATION (use previous questions if household head is same person)**

1. What does your typical day look like/ daily activities?
   1. Probe: livestock, other chores, where to get water/ feed
   2. Prompt: have these activities changed during displacement?
2. What is your main worry?

**2. DISPLACEMENT**

1. Can you tell me about your (displacement) experiences and challenges? You moved here from quite a long way away, what was that like? How difficult was it?
   1. Prompt: choice of destination, transport, family members, assets (animals), main issues
   2. Probe: have you been displaced before? can you describe that experience and whether it was similar/ difference?
2. How has the displacement affected you and your family? How has moving here affected you? What about your family? Would you say they are happy with the new situation?
   1. Prompt: resources, environment, shelter, nutrition, income generation of livestock vs non-livestock income sources before and after displacement, spending priorities, animal handling, grazing, slaughter, health
   2. Probe: How do you address these changes? Examples.
3. Can you tell me about your animals and how their situation changed due to the displacement? In terms of livestock, what animals did you have before and what you have now? Is it very different? How did it impact your life?
   1. Probe: resources, space, facilities, electricity, water, site, numbers
   2. Probe: which animals do you look after, since when, why these types of animals, where to keep/ shelter (depend on species?), use of animals (changes?), health, births/ deaths
   3. Probe: When and how did you lose livestock?
   4. Prompt: death, sales, slaughtered, other?

**3. VULNERABILITY AND RISK**

1. What is the current status of your/ household members health?
   1. Prompt: zoonotic diseases
   2. Probe: change during displacement, concerns, how is the sickness hurting you?
2. What do you think puts people at risk of diseases?
   1. Prompt: (political, environmental, economic, lifestyle, slaughter, cooking, etc) risk factors [animals]
   2. Probe: Example of last displacement (perceptions, assumptions), actions of community
3. Do you visit health services? Why (not): location, expense
   1. Prompt: When do you decide to seek healthcare? previous experience, word of mouth, funds (threshold)
   2. Probe: Which specific facilities? [compare with local health data?]
   3. Probe: Do you use medication, from what source, how much do you spend on these?
4. Can you describe animal illnesses and how do these impact the household?
   1. Prompt: severity, symptoms
   2. Probe: Why do you think the animals are more/ less deceased (causes)?
5. What do you do with your animals if/ after they are sick (example last time an animal was sick).
   1. Prompt: vaccination, veterinary (or why not?); and if that does not help?
   2. Probe: Do you report on disease?
   3. Probe: Where do you get your information? How do you know what to do?

NB: If no animals: where are they and what could have been done so you could keep the animals?

**4. SERVICES**

1. Have you received any [humanitarian] assistance? What sort of help have you received? From whom? Who helped the most?
   1. Prompt: type, from whom (including solidarity); sufficient?
   2. Probe: Can you tell me what other services you access and how?

**5. FUTURE**

1. Can you tell me what your personal/ family plans are? What will you do in the future?
   1. Prompt: stay here, return, move on?
   2. Probe: what would influence decision?
   3. Prompt: Policies, assistance, services [prevent protracted displacement]
2. Is there anything that you feel I should have asked you or that you want to tell me in relation to this issue?

With many thanks for your help
